# Chemical Composition, Antimicrobial, Antioxidant, and Anticancer Activities of *Jacquemontia pentantha* Essential Oils

**DOI:** 10.3390/molecules31020296

**Published:** 2026-01-14

**Authors:** Noorah A. Alkubaisi, Mashail Fahad Alsayed, Hissah Abdulrahman Alodaini, Fuad Alanazi, Abdulhadi M. Abdulwahed, Ibrahim M. Aziz

**Affiliations:** 1 Department of Botany and Microbiology, College of Science, King Saud University, Riyadh 11451, Saudi Arabia; malsayed@ksu.edu.sa (M.F.A.); halodaini@ksu.edu.sa (H.A.A.); 2 Department of Clinical Laboratory Sciences, College of Applied Medical Sciences, King Saud University, Riyadh 12372, Saudi Arabia; foalanazi@ksu.edu.sa (F.A.); aabdulwahed@ksu.edu.sa (A.M.A.)

**Keywords:** medicinal and aromatic plant resources, essential oil, GC–MS analysis, plant extracts, bioactivity, application

## Abstract

*Jacquemontia pentantha* (Jacq.) G. Don. (*Convolvulaceae*): This is a plant with rich ethnobotanical uses, but its essential oil (EO) composition and overall biological properties remain largely uninvestigated. In this research, the *J. pentantha* EO (JPEO) is characterized in a thorough manner, with an evaluation of its in vitro antioxidant, antimicrobial, and cytotoxic properties, aiming to provide scientific support for ethnobotanical uses, as well as the definition of new potentialities. The EOs were isolated from the aerial part of the plant via hydrodistillation, and a qualitative analysis of the components was carried out via GC–MS. The biological properties were investigated by means of standard in vitro assays: namely, DPPH and ABTS for the measurement of antioxidant activity, the disk diffusion technique, and the microbroth dilution assay for the evaluation of antimicrobial activity against six bacterial species, as well as for the assessment of the activity against five species of *Candida* fungi, whereas the cytotoxic activity against MCF-7 and HepG2 cells was assessed using the MTT assay. Preliminary characterization of the EOs via GC/MS revealed a particular “chemical profile” with a high concentration of himachalene-type sesquiterpenes, namely, β-himachalene (6.47%) and (+)-α-himachalene (6.46%), together with phenolic monoterpenoids. The EOs showed significant antioxidant activity (IC_50_ = 172.41 and 378.94 µg/mL, respectively), high phenolic content (97.34 mg GAE/g), and significant antibacterial activity (MIC = 4.68 µg/mL), especially against *Pseudomonas aeruginosa*, as well as against *Candida albicans* (MFC = 3.90 µg/mL), together with dose-dependent cytotoxic effects on the two cell lines, with IC_50_ = 161.62 and 151.87 µg/mL, respectively. This research indicates that the EO of this plant is a potential source of a certain “chemical profile” with noteworthy antibacterial and cytotoxic properties, thus providing scientific support for its ethnobotanical use and highlighting its particular potential for developing pharmaceutical agents against infections and cancer.

## 1. Introduction

Explorations in nature, particularly of aromatic and medicinal flora, for their potential bioactive chemicals, are being pursued in scientific circles. Essential oils (EOs) are complex mixtures of volatile secondary metabolites that are valuable in various fields, such as pharmaceuticals, cosmetics, and the food industry, because of their wide-ranging biological properties, which include antibacterial, antioxidant, and anti-inflammatory activities [1]. Whereas many studies have focused on established species of families such as *Lamiaceae*, for example, *Mentha piperita*, there is significant biodiversity within other plant families that has not yet been explored and therefore provides a fertile frontier for the discovery of medicinal compounds [2,3].

*Jacquemontia pentantha* (Jacq.) G. Don, also known as sky blue clustervine and belonging to the *Convolvulaceae* family [4], represents a candidate worthy of further in-depth phytochemical and pharmacological investigation. It is notable for its delicate growth habit and dense clusters of bright, true blue flowers that bloom frequently through fall and winter, providing a rich nectar source for pollinators. This profile places *J. pentantha* in an ecologically diverse family; however, it has a unique phytochemical composition, especially its EO composition, which is not well documented compared with that of other aromatic species [5].

*J. pentantha* is native to coastal dunes and hammocks in Florida, USA, and the Caribbean, and is cultivated as an ornamental plant in arid and semi-arid regions, including Saudi Arabia [6]. Its adaptability to sandy, well-drained soils, as well as exposure to intense sunshine and drought stress, has been shown to alter secondary metabolite production in plants [6]. No specific ethnomedicinal information about *J. pentantha* was found within the literature reviewed for this manuscript, but related ethnobotanical research illustrates the worldwide use of plants from the family *Convolvulaceae* within traditional medicinal systems. In support of this, quantitative ethnobotany studies in regions such as the caatinga of Brazil and inventories from southern Africa document medicinal uses of native species, which illustrates the strong coupling between biodiversity and traditional healthcare knowledge [7]. This context strengthens the rationale supporting the study of *J. pentantha* because related species within its family and region often share bioactive potential.

The biological activities of *J. pentantha* EO (JPEO) can be postulated by drawing parallels with other well-investigated EOs, such as *Mentha piperita*. Peppermint EO is reported to possess high antibacterial activity, especially against Gram-positive bacteria, as well as antioxidant, anti-inflammatory, and wound-healing activities, due to its components such as menthol and menthone [8,9,10]. Additionally, studies on EOs obtained from distinct species have indicated that their biological activity is strongly related to their chemical composition, which in turn can be modified according to the region, part of the plant, and form of extraction [11]. Hence, the study of JPEO would provide a detailed analysis necessary for the chemical description of its volatile profile; scientific assessment of its potential antioxidant, anti-inflammatory, and antibacterial actions in vitro and in animal models; and its safety and eventual synergistic effects [9].

Several factors make JPEO a high-potential candidate. EOs are known for their rich bioactive compounds with proven antibacterial, antioxidant, and anticancer activities, at least partially justifying them as objects of interest for novel therapies or natural additives [12]. Although some studies have focused on crude solvent extracts of *J. pentantha*, which highlight the presence of important phytochemical classes, such as phenols and flavonoids [4,5,13], with their corresponding bioactivities, the chemical profile of its EOs, which are sometimes quite distinct and rich in very active compounds, is still unknown. A clear strategy for the study of such plants is already available on the basis of the combination of phytochemical analysis and a battery of biological assays, thus representing a robust blueprint for this work, which, moreover, is highly relevant since it fits into the global push toward the documentation of biodiversity and the discovery of new bioactive natural products. Therefore, the current study aims to explore, for the first time, the biological potential and underlying phytochemical basis of JPEO. Specifically, this study aims to chemically characterize JPEO by means of Gas Chromatography–Mass Spectrometry analysis, assess its in vitro antimicrobial activity against selected bacterial and fungal pathogens, determine its antioxidant capacity through free radical scavenging assays, and investigate its in vitro cytotoxic effects on a panel of human cancer cell lines.

## 2. Results

### 2.1. Extraction Yields

The extraction yield, calculated based on dry matter weight (*w*/*w*), demonstrated that the JPEO extraction process achieved a yield of 2.76 ± 0.23% (*v*/*w*).

### 2.2. Chemical Composition of JPEO

**Figure 1:**
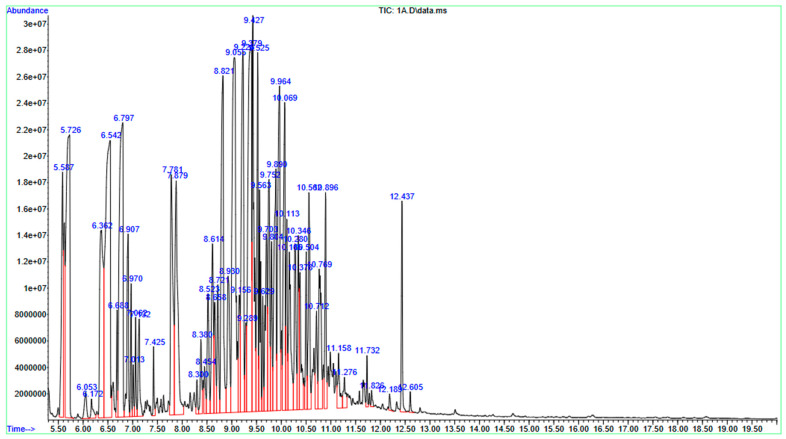
Gas Chromatography–Mass Spectrometry chromatogram of *Jacquemontia pentantha* essential oil. The main component of the extract is indicated by a big peak, and all spectral peaks correlate with recognized compounds.

The chemical composition of the JPEO was ascertained using GC-MS. Seventy-three chemicals were found during the GC-MS investigation. By comparing the compounds’ retention durations (RT), relative peak areas (%), molecular formulas (MF), and molecular weights (MW) to those of reference compounds found in the NIST mass spectral database, the compounds were tentatively identified. α-Thujone was the main component of EOs, accounting for 6.72% of the total. β-Himachalene (6.46%), (+)-α-himachalene (6.45%), (+)-2-bornanone (5.31%) and eucalyptol (5.26%) (Table 1 and Figure 1).

### 2.3. Total Phenolic Content (TPC) and Total Flavonoid Content (TFC) of the JPEO

TPC (97.34 ± 2.11 mg GAE/g of extract; R^2^ = 0.912) was found to be more abundant in the TPCs and TFCs of JPEO than TFC (47 ± 1.42 mg QE/g of extract with R^2^ = 0.941).

### 2.4. 1,1-Diphenyl-2-picryl Hydrazyl (DPPH) and 2,2′-Azino-Bis (3-Ethylbenzothiazoline-6-Sulfonic Acid) (ABTS) Scavenging Assay

**Figure 2:**
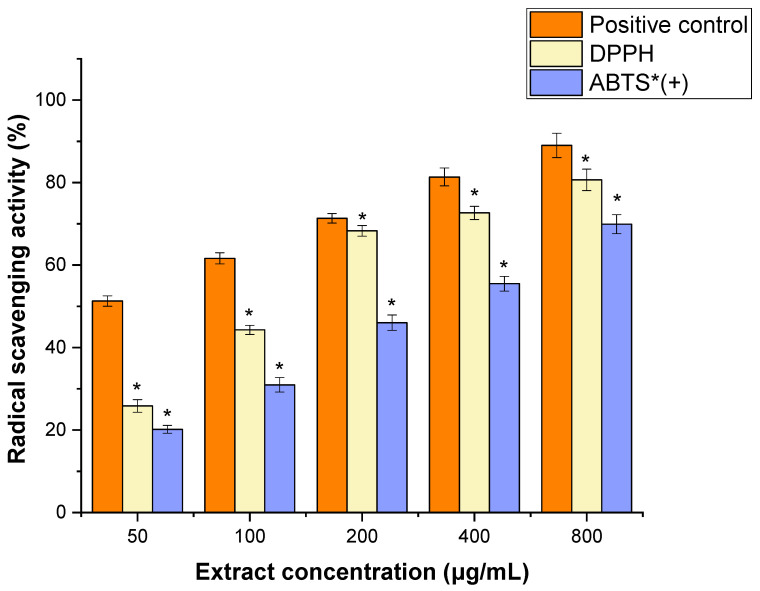
1,1-Diphenyl-2-Picrylhydrazyl and 2,2′-Azino-bis(3-ethylbenzothiazoline-6-sulfonic acid) radical scavenging activity of *Jacquemontia pentantha* essential oil. The scavenging activity of *Jacquemontia pentantha* Essential Oil was significantly lower (*) than that of the positive control as determined by Student’s *t*-test, at a significance level of *p* < 0.05. + denotes a radical cation.

The antioxidant properties of JPEO were evaluated using two different assays: DPPH and ABTS radical scavenging activities. JPEO showed moderate scavenging activity for DPPH (IC_50_ value: 172.41 ± 3.15 μg/mL) and ABTS radicals (IC_50_ value: 378.94 ± 2.18 μg/mL) as compared to the positive control (IC_50_ value: 153.11 ± 1.14 μg/mL) (Figure 2).

### 2.5. Antibacterial Effects of JPEO

The disk diffusion test and the traditional broth microdilution technique were used to assess the antibacterial activity of JPEO against six pathogen strains. Table 2 summarizes the findings of measuring the diameter of the zone of inhibition (ZOI), as well as the minimum inhibitory concentration (MIC) and minimum bactericidal concentration (MBC) values. Compared with chloramphenicol (25 μg/mL), a positive control, the majority of bacteria were sensitive to the JPEO (*p* < 0.05). The JPEO had MIC values ranging from 6.25 ± 0.00 to 25 ± 0.00 μg/mL for Gram-positive bacteria and from 4.68 ± 2.21 to 25 ± 0.00 μg/mL for Gram-negative bacteria. *Pseudomonas aeruginosa* had the strongest antibacterial activity, with an MIC of 4.68 ± 2.21 μg/mL.

### 2.6. Antifungal Activity of JPEO

The MIC and minimum fungicidal concentration (MFC) values of JPEO against the yeasts under investigation ranged from 7.81 ± 3.51 to 125 ± 11.61 μg/mL and 7.81 ± 0.88 to 250 ± 9.98, respectively. The antifungal efficacy of JPEO exhibited a gradual increase with rising concentrations; at 125 and 1000 μg/mL, the ZOI started to rise significantly (*p* < 0.05), although not as much as the positive control (25 µg/mL of fluconazole). The highest MIC (7.81 ± 0.88) and MFC values (3.90 ± 0.68) were observed for *C. albicans.* However, JPEO was less effective against *Candida glabrata* in terms of the MIC (250 ± 9.98 μg/mL) and MFC (125 ± 11.61 μg/mL). No ZOIs were detected in the negative control, which contained 0.01% DMSO with SDB (Table 3).

### 2.7. Cell Cytotoxicity

**Figure 3:**
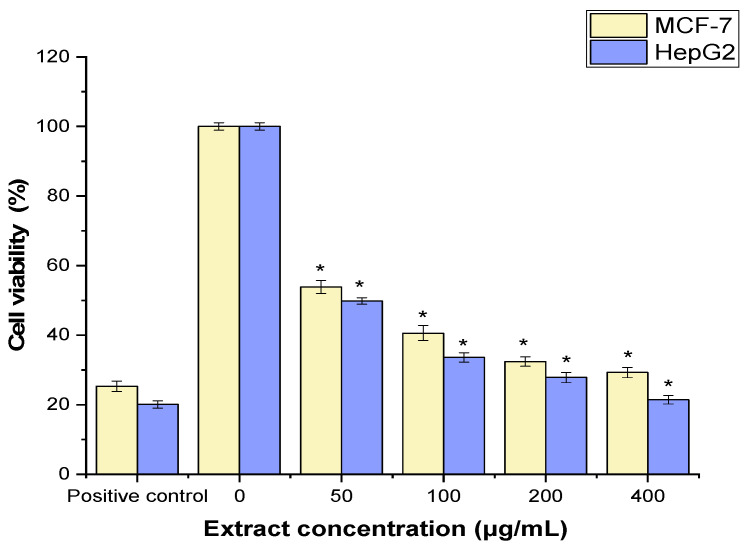
Anticancer effects of *Jacquemontia pentantha* essential oil on MCF-7 and HepG2 cells. Statistical analysis was performed using Student’s *t*-test. The mean ± SD for the triplicate is shown (* = *p* < 0.05 relative to the untreated cells (negative control)).

The MTT assay was used to assess the anticancer properties of JPEO against the cancer cell lines MCF-7 and HepG2. As shown in Figure 3, JPEO significantly suppressed cancer cells in comparison to a positive control (cisplatin 30 µg/mL) (*p* < 0.05). JPEO inhibited the proliferation of MCF-7 (IC_50_ = 161.62 ± 1.92 μg/mL) and HepG2 cells (IC_50_ value of 151.87 ± 2.15 μg/mL).

## 3. Discussion

The volatile compound profile analysis of JPEO revealed a chemotaxonomically distinctive volatile compound profile. JPEO has a high diversity of sesquiterpene molecules, which are the most abundant (45.1%), followed by monoterpene molecules (30.4%). These results align with recent reports, which revealed that the phytochemical analysis of different extracts of *J. pentantha* revealed a constant composition of terpenoids, carbohydrates, phenolics, alkaloids, flavonoids, and steroids [4,13]. The different compositions of the chloroform–methanol extracts of the aerial parts of *J. pentantha* revealed the presence of palmitic acid, Phytol, and β-amyrin as the major constituents [5]. This finding is highly relevant from a chemotaxonomic standpoint, given the high level of curiosity for a species with the potential to produce specialized compounds with specific biological features.

The most characteristic aspect of JPEO is, therefore, the high relative abundance of himachalene-type sesquiterpene hydrocarbons, particularly β-himachalene (6.47%) and (+)-α-himachalene (6.46%). These molecules are generally uncommon as dominant components in the EOs of the more commonly investigated *Convolvulaceae* species, such as *Ipomoea* [15]. This finding implies that JPEO possesses a distinctive chemical signature compared to other species in the same plant family. However, a recent study revealed that the main volatile compounds of the leaf extract of *Convolvulus arvensis* (*Convolvulaceae*) were rich in cuprenne, thymol, himachalene, and longifolene [16]. On the other hand, heachalene isomers are characteristic molecules of cedarwood oils, particularly those from *Cedrus atlantica* (Atlas cedar), which is endemic to North Africa [17]. The preponderance of these sesquiterpenoids in JPEO might, therefore, imply convergent ecological adaptation linked to resistance to a particular environment, which brings the chemical defense strategy of this particular vine closer to that of a long-lived desert tree species rather than to other herbaceous plants. This himachalene-dominant profile differs from observations on other *Convolvulaceae* species, where these chemicals are often minor elements or where the dominating volatiles are from other groups, such as aliphatic hydrocarbons, common monoterpenes, or other sesquiterpene types [4,5,13].

Additionally, JPEO has marked oxygenated monoterpenoids with established known effects and properties. Notably, α-thujone (6.72%), camphor (1.41%), and aka (+)-2-bornanone (5.31%) were detected. The presence of eucalyptol (5.26%) aligns JPEO with other Mediterranean plants used for medicine, particularly Salvia species, which are recognized for their variety of qualities, including their effects on respiration [17,18]. The presence of thymol (3.31%) and carvacrol (2.79%), phenolic monoterpenoids from thyme and oregano EOs with pronounced antimicrobial properties, synergizes with monoterpenes, indicating a synergistic effect on microorganisms, which can pivot toward targeted pharmacological properties [4]. Last, the high percentage of α-thujone requires critical safety considerations. However, it has known neurotoxicity; it has been shown to have anticancer, antidiabetic, and antimicrobial activities [19]. This warrants a cautious assessment of the oil’s biological potential. Any further development would necessitate extensive toxicological studies to determine safe dosage limits, fractionation procedures to separate the beneficial himachalene/phenolic fractions from thujone, or its use only in non-systemic, topical treatments where systemic absorption is low.

Within the broad landscape of pharmacology across *Convolvulaceae*, this JPEO profile presents a familiar mixture but also introduces some fresh notes. In related species, such as *Ipomoea asarifolia*, known polar compounds are flavonoid-like rutin and phenolics such as chlorogenic acid, which may act as strong anti-inflammatory agents [20]. However, in *J. pentantha*, a different angle is presented with its volatile and terpene-rich signature, broadening that perspective. Its *EO* is marked by β-caryophyllene (4.06%) and related compounds (2.59%), which indeed exhibit anti-inflammatory, antibacterial, and analgesic activities [21,22,23]. This particular volatile fraction likely underpins much of the traditional use of Convolvulaceae in folk medicine, alongside water-soluble metabolites [24].

When the nonvolatile polar compounds in JPEO were quantified, the TPC was 97.34 ± 2.11 mg GAE/g extract, whereas the TFC was 47 ± 1.42 mg QE/g. The calibration curves showed strong linearity, with R^2^ = 0.912 and 0.941, respectively, highlighting the reliability of these spectrophotometric measurements. In simple terms, JPEO contains a high load of phenolic antioxidants, which are approximately twice as abundant as flavonoids. This signals a rich reserve of nonvolatile, polar phenolics that GC–MS would miss if we were only scanning for volatiles. The traditional anti-inflammatory and wound-healing uses of *J. pentantha* likely arise from a synergistic mixture of volatile terpenoids, such as antibacterial thymol and carvacrol, with these potent phenolic antioxidants [25]. This pattern fits with the wider role of phenolics and flavonoids in the traditional therapeutic effectiveness of plants, such as those employed in Saudi Arabia [26].

Similar observations were reported by a recent study reported that the total flavonoid content reached 50.18% in hexane and 87.05% in ethyl acetate leaves [13]. In contrast, Dhokiya et al. (2025) reported much lower TPCs (6.3%) and TFCs ranging from 3–6% across various extracts and plant parts, which illustrates significant methodological differences [4]. Eskander et al. (2019) focused their interest on crude methanol aerial-part extracts and identified numerous flavonoids and phenolic acids via LC–MS/MS [5]. Although they confirmed those phenolic classes qualitatively, no direct TPC/TFC numbers were given for comparison. A recent study on *Ipomoea asarifolia* attributed its excellent anti-inflammatory action to phenolics such as chlorogenic and caffeic acids, which are very common in plant extracts [27]. The significant TPC and TFC values, even though relatively high for an EOs, likely represent the presence of phenolic monoterpenoids (e.g., thymol, carvacrol) and perhaps co-distilled semi-volatile phenolic compounds observed in the GC-MS study, which are recorded by the colorimetric assays [25].

JPEO demonstrated moderate antioxidant activity against DPPH and ABTS radicals, with IC_50_ values of 172.41 ± 3.15 μg/mL and 378.94 ± 2.18 μg/mL, respectively. This trend is in line with the existing understanding of the limitations of antioxidant assays for complex matrices such as EOs and contributes to the framing of the bioactivity of the oil against its unique chemistry [28]. It is not unusual to find that the two IC_50_ values differ: DPPH operates on a HAT principle and favors strong hydrogen-donating antioxidants, whereas ABTS uses a SET mechanism and therefore responds to a wide range of antioxidants, including both lipophilic and hydrophilic ones [28,29]. Previous studies reported higher DPPH (450 ± 17 μg/mL) and ABTS (538 ± 27 μg/mL) IC_50_ values for *J. pentantha* leaves [13]. The antioxidant activity of JPEO can be contextualized as moderate compared to highly antioxidant EOs like *C. arvensis* and *Ipomoea pescaprea*, which often exhibit IC_50_ values below 50 µg/mL in DPPH assays [16]. However, it is comparable to or superior to many other plant EOs used in aromatherapy and traditional medicine [30]. This level of activity may help to reduce oxidative stress in situations where the oil is applied topically or in controlled formulations. The antioxidant behavior of JPEO could reasonably be attributed to its key components: sesquiterpene hydrocarbons (such as himachalenes and caryophyllene) combined with oxygenated monoterpenoids (thujone, eucalyptol, thymol), which would cumulatively enhance antioxidant defense [31].

JPEO showed significant antibacterial activity against all tested Gram-positive (*S. aureus*, *S. epidermidis*, *E. faecalis*) and Gram-negative organisms (*E. coli*, *K pneumoniae*, *P. aeruginosa*). The strongest activity was reported against *P. aeruginosa*, a notable opportunistic pathogen typically associated with antibiotic resistance [32], with the lowest MIC value of 4.68 ± 2.21 µg/mL and an MBC of 6.25 µg/mL. This is an important finding, as *P. aeruginosa’s* low-permeability outer membrane and efflux pump systems make it less vulnerable to plant extracts [33]. This antibacterial action is consistent with the established antimicrobial hierarchy of EO components, with phenolic substances (such as thymol and carvacrol) being among the most active. The presence of these phenolics in the chemical profile of JPEO, as evidenced by the notably high TPC and TFC values, is expected to play a significant role in its mechanism of action, which normally includes the rupture of microbial membranes [34]. Similar findings were previously reported for the *J. pentanthos* leaf extract against *S. aureus* (MIC = 25 µg/mL), whereas, in contrast to our findings, it did not affect *E. coli* [4], which may be due to the fundamental difference in extraction methods. Additionally, the distinct set of volatile terpenoids and phenolic monoterpenes (such as thymol and carvacrol) explains their high effectiveness at disrupting microbial membranes [35]. The *EOs* of *Mentha piperita* L. (*Convolvulaceae*) exhibited similar activity against *S. aureus* (MBC = 3.7 μg/mL) and *Listeria monocytogenes* (MBC = 7.43 μg/mL) [36]. Additionally, *Convolvulus* arvensis exhibited strong antibacterial activity against *K. pneumoniae* (MIC = 21.35 µg/mL) and *S. aureus* (MIC = 28.62 µg/mL) [16]. Additionally, a recent study found that eleven *Convolvulaceae* species’ ethanolic extracts have varying antibacterial activity against *S. aureus* (ZOI = 8–12.2 mm), *B. subtilis* (ZOI = 9–13.3 mm), *E. coli* (ZOI = 4.4–11.3 mm), and *P. aeruginosa* (ZOI = 7.4–13.3 mm) [37]. This comparison demonstrated the great antibacterial potential of *J. pentantha* as an underexplored species. Phenolic monoterpenoids such as thymol and carvacrol are well-known for their capacity to disrupt microbial membranes by integrating into the lipid bilayer, increasing permeability, and dissipating the proton motive force, causing cell death [34]. The high content of sesquiterpene hydrocarbons, including himachalenes and β-caryophyllene, and oxygenated monoterpenes might also contribute to the antibacterial activity by affecting membrane integrity and fluidity, allowing other bioactive components to work more effectively [35]. EOs have a significant benefit over single-target antibiotics because of their multitarget mechanism, which reduces the likelihood of bacterial resistance development [38].

The antifungal experiment demonstrated a broader spectrum of action against *Candida* species. JPEO was highly effective against *C. albicans*, with a low MIC of 7.81 ± 0.88 µg/mL and an MFC of 3.90 ± 0.68 µg/mL. This high efficacy against the most common human fungal infection is quite encouraging. The oil was less effective against *C. glabrata* (MIC: 250 µg/mL), a species recognized for its lower susceptibility to antifungals. This disparity demonstrates species-specific sensitivity to EO components. Like antibacterial activity, antifungal activity is most likely mediated by lipophilic terpenoids and phenolics in JPEO, which can integrate into the fungal cell membrane, compromising its integrity and causing cellular contents to seep out [39]. An MFC lower than the MIC indicates a strong and quick fungicidal impact against this species, which is considered significant activity [40]. Despite the previously reported antifungal activities of JPEO, some studies highlighted similar effects for other *Convolvulaceae* members. An early study reported that *C. arvensis* EO exhibited important antifungal activity against *Fusarium oxysporum* (MIC = 18.7 g/mL) and *C. albicans* (MIC = 19.4 g/mL) [16], whereas the volatile fractions of *C. althaeoides* L. roots promoted antibiofilm formation by *C. albicans* (0.87 mg/mL), *C. glabrata* (1.75 mg/mL), and *C. tropicalis* (0.87 mg/mL) [41]. Additionally, in the recent study, *Convolvulaceae* members showed antifungal potential against *Aspergillus niger* and *Aspergillus oryzae* [37]. Furthermore, the aqueous extract of the leaves of *Ipomoea batatas* has antifungal potential against *Malassezia furfur* (ZOI = 30.8 mm) [42]. The species-specific variation in efficacy may be attributable to changes in the cell membrane ergosterol concentration or the performance of efflux pumps between *Candida* species, which can impact the accumulation and activity of these oil components [43]. While the MIC/MFC values are pharmacologically interesting, more formulation studies and toxicity assessments are necessary to establish attainable therapeutic doses and safety limits.

JPEO was tested via the MTT assay and was found to have substantial cytotoxic activity against two human cancer cell lines, MCF-7 (breast adenocarcinoma) and HepG2. JPEO inhibited growth in a dose-dependent manner, with half-maximal inhibitory concentration (IC_50_) values of 161.62 ± 1.92 and 151.87 ± 2.15 µg/mL, respectively. Although the IC_50_ values are higher (indicating lower potency) than those of the standard chemotherapeutic agent cisplatin (used here at 30 µg/mL), they represent meaningful baseline cytotoxic activity for a crude *EO* and provide a critical foundation for understanding the plant’s pharmacological potential. The cytotoxic action of JPEO is rationally linked to its distinctive chemical signature, which has been established through previous research. The oil is not characterized by a single, very effective anticancer component but presumably exerts its effects through the combined and possibly synergistic action of several ingredients, such as thymol, carvacrol, Himachalenes, and β-caryophyllene [17,44]. Similarly, the EO of *C. lanatus* displayed substantial cytotoxic activity against a HepG2 cell line with an IC_50_ of 38.93 µg/mL, an order of magnitude greater than the JPEO data reported in this study [44]. Additionally, *C. galaticus* has anticancer potential against MCF-7 cells, with an IC_50_ of 0.32 μg/mL [45]. This finding demonstrates significant variation in bioactive potential even within the same plant family, most likely owing to variances in specific chemical profiles. Eskander et al.’s 2019 study on *J. pentantha* methanol extracts likewise revealed cytotoxic activity, indicating that the plant has anticancer properties [5]. The discovery that the volatile *EO* has cytotoxic activity greatly increases the known bioactive fractions of this species. Owing to its unique terpenoid-rich volatile profile, JPEO adds a new chemical profile with cytotoxic characteristics to the family’s pharmacological repertoire.

The current study is considered the first comprehensive evaluation of JPEO, setting a distinct profile that is marked by a high amount of himachalene-type sesquiterpenes, which are uncommon in the *Convolvulaceae* plant family. This research contributes to a cohesive profile that is different from past research on crude solvent extracts of plants with respect to measuring the actual bioactivity of hydrodistilled EOs. These findings are consistent in that it has potent antibacterial properties, especially against challenging bacteria such as *P. aeruginosa* and *C. albicans*, as well as against human cancer cells.

However, some inherent limitations are due to the in vitro nature of this study, but not necessarily its efficacy or safety in living systems. For verification, the retention times of key, it is necessary to utilize linear interpolation concerning the retention lengths of two common n-alkane mixes (C_8_–C_20_ and C_21_–C_40_) to calculate the retention indices of the components. It is important to emphasize that this study focuses on the chemical and biological profile of JPEO from a specific harvest and geographical region. EO content is known to fluctuate depending on seasonal, climatic, edaphic, and genetic factors [11]. As a result, the repeatability of this unique himachalene-rich profile must be validated by analyzing multiple samples obtained throughout different seasons from diverse cultivated or wild populations. Such research would determine whether the chemical signature of the species in this location is consistent or varied. The presence of neurotoxic α-thujone requires thorough safety testing and may limit usage to topical formulations with little systemic absorption. The current study focused on demonstrating cytotoxic activity as a preliminary screen for anticancer potential. However, the absence of parallel testing on normal cell lines (such as normal breast epithelial cells MCF-10A, normal hepatocytes, or peripheral blood mononuclear cells) prevents determination of selective cytotoxicity. Selectivity index (SI = IC_50_ normal cells/IC_50_ cancer cells) is a critical parameter for therapeutic evaluation, with SI > 3 generally considered promising for anticancer agents. Future studies should include: (1) normal cell line counterparts (MCF-10A for MCF-7 comparison; normal hepatocytes for HepG2 comparison). In addition, although potential toxicity against cancer cells has been observed, the fact that the research has not been conducted on noncancer cell lines hampers the measurement of the degree of selectivity, which is extremely vital for a therapeutic agent. The characterization of chemicals is accurate but is based on one specimen that fails to consider a degree of variability owing to the environment. Therefore, future studies are needed to elucidate the mechanisms behind the observed antibacterial and cytotoxic properties. It is essential to evaluate the selectivity of JPEO by determining the level of its cytotoxicity on normal human cell lines, hence providing a therapeutic index. Studies on potential synergistic effects with conventional medications might enhance the potency against resistant bacteria. Later, in vivo toxicity and efficacy studies are vital in the development stages, especially in appropriate models used for preclinical assessment. In terms of plant metabolites, the isolation of essential active components, such as isomers of himachalene, to define individual properties and the development of new formulations, such as nanoemulsions with the goal of increasing oil stability, availability, and potential uses, are pertinent.

## 4. Materials and Methods

### 4.1. Preparation of EOs

The J. pentantha leaf was obtained from the El-Kharj region, Saudi Arabia, in August of 2025. The selected plant species were validated by Prof. Dr. Mohammed Fasil, and voucher specimens were deposited in the herbarium of the Department of Botany and Microbiology, College of Science, King Saud University, Riyadh, Saudi Arabia (KSU NO-14307). The leaves of *J. pentantha* were meticulously washed with distilled water, then air-dried at room temperature before being processed into a powder with an electric mixer. As previously mentioned [46], the volatile oils of J. pentantha leaf extraction were extracted by hydrodistilling 100 g for three hours with 1000 mL of distilled water via a Clevenger-style device. Finally, the obtained oils were desiccated via anhydrous Na_2_SO_4_. The extraction was performed in triplicate. The extract yield% was calculated via the following formula: yield (%) = weight of solvent-free extract (g) × 100/dried extract weight [47]. The pooled EOs from the three extractions were stored in sealed amber vials at 4 °C under a nitrogen atmosphere for further analysis within 2 weeks of extraction.

### 4.2. GC-MS Analysis

The volatile JPEO was analyzed on a gas chromatography–mass spectrometry (GC–MS) system coupled to an Agilent 5977A MSD system (Agilent Technologies Inc., Santa Clara, CA, USA). For GC–MS analysis of the extracts, an Agilent GC 7890A instrument was used, which was combined with a triple-axis detector (5975 °C) with a single quadrupole mass spectrometer. An Agilent HP 5MS column (30 m × 0.25 mm × 0.25 µm film thickness) was used as the chromatographic column, and high-purity helium was used as the gas carrier at a flow rate of 1 mL/min. The temperature of the injector was 280 °C, and it was equipped with a splitless injector at a ratio of 20:1. The MS source temperature was 230 °C, and the Quad temperature was 150 °C. The oven temperature, which was initially set at 40 °C (held for 1 min), was gradually increased to 150 °C at 5 °C min^−1^ (held for 1 min). The temperature was increased further to 300 °C at 5 °C min^−1^ for 1 min. The temperature of the MS ion source was set at 150 °C, and 280 °C was selected as the inlet line temperature. The solvent delay was three minutes, and the scan range was set at 40–600 mass ranges at 70 eV electron energy. Unknown compounds were discovered by matching the spectra with those from the National Institute of Standards and Technology library (NIST 2008), which is part of the system. The total time required for analyzing a single sample was approximately 55 min. The MF and MW reported for the identified compounds were retrieved from reference databases PubChem [14], rather than being experimentally determined in the present work.

### 4.3. Determination of the TPC and TFC

The Folin–Ciocalteu method, which was detailed in an earlier paper [48], was used to compute the TPC. Once the extract concentration was determined, 0.25 mL of the extract was combined with 1.25 mL of the Folin–Ciocalteu reagent (phosphomolybdate and phosphotungstate) and 7.5% NaHCO_3_ to create a combination. After this mixture was incubated for 15 min at 45 °C, the absorbance at 765 nm was measured via a spectrophotometer (U2001 UV–vis Spectrophotometer, Hitachi, Tokyo, Japan). The results were expressed as gallic acid equivalent (mg GAE)/g of extract.

The TFC was determined via the aluminum chloride (AlCl_3_) colorimetric method, as described previously [49]. Using this approach, 0.1 mL of each extract was separately incubated for 30 min at room temperature with 2.0 mL of a 2% AlCl_3_ solution and 1.0 M sodium acetate. A spectrophotometer (U2001 UV–vis Spectrophotometer, Hitachi, Japan) calibrated at 420 nm was used to evaluate the extract’s absorbance after incubation. The unit of measurement for the TFC is mg quercetin equivalent mg (QAE)/g of extract.

### 4.4. Antimicrobial Activity

#### 4.4.1. Collection of Microbial Strains

The antimicrobial properties of JPEO were evaluated against three Gram-positive bacteria, *S. aureus* (ATCC-23235), *S. epidermidis* (ATCC-12228), and *E. faecalis* (ATCC-29212); and three Gram-negative bacteria, *E. coli* (ATCC-25922), *K. pneumoniae* (ATCC-13883), and *P. aeruginosa* (ATCC-27853), were used. The selected fungal strains were *C. albicans* (ATCC-10231), *C. glabrata* (ATCC-2001), *C. kefyr* (ATCC 66028), *C. parapsilosis* (ATCC-22019), and *C. tropicalis* (ATCC-750).

#### 4.4.2. Antibacterial Activity of JPEO

The agar well diffusion method was used to determine the antibacterial potential of JPEO against selected bacterial strains according to [50]. A bacterial suspension from a pure isolate of each selected bacterial strain was cultured and incubated overnight at 37 °C on nutrient agar plates, after which one hundred microliters (1.0 × 10^7^ bacterial CFU/mL) of each bacterial suspension was moistened and spread onto nutrient agar plates. Six wells (5 mm diameter) were made in agar plates via a sterile cork borer. The wells were completely filled with various concentrations of JPEO (125, 250, 500, and 1000 μg/mL; 100 µL/well) and subsequently left for 30 min at 4 °C to allow proper diffusion of the JPEO into the agar. The agar plates were then incubated at 37 °C overnight. The ZOI around the JPEO-containing well was checked, and the diameter of the ZOI was measured via a measuring ruler. Chloramphenicol (25 μg/mL) and 1% DMSO in nutrient broth were used as positive and negative controls, respectively.

#### 4.4.3. Determination of the MIC and MBC of JPEO

The MIC and MBC of JPEO were evaluated against *E. coli* (ATCC-25922), *Klebsiella pneumoniae* (MTCC-13883), and *P. aeruginosa* (MTCC-27853); and three Gram-positive *S. aureus* (ATCC-23235), *S. epidermidis* (MTCC 12228), and *E. faecalis* (ATCC-29212), via the broth microdilution method in 96-well microplates according to [51] with some modifications. First, JPEO was diluted in nutrient broth with 1% DMSO (to concentrations ranging from 1.95 to 1000 μg/mL, 100 μL) and added to each well. The bacterial suspension (5 × 10^6^ CFU/mL, 10 µL) was subsequently added to each well. Chloramphenicol (25 μg/mL) was used as a positive control, whereas 1% DMSO with nutrient broth was used as a negative control. After 24 h of incubation at 37 °C, the plates were visually examined for bacterial growth. Following treatment, 20 mL of triphenyl tetrazolium chloride (TTC) (2 mg/mL in PBS) was then added to each well. After incubation for 20 min at 37 °C, the MIC was determined. The well containing the lowest concentration of the colorless solution was interpreted as the MIC, whereas the lowest concentration of JPEO at which no growth occurred was determined as the MBC [52].

#### 4.4.4. Determination of the Antifungal Activity of JPEO

Preliminary screening of the antifungal activity of JPEO was performed against selected yeasts through an agar well diffusion assay according to a previous study [53] with some modifications. Briefly, five selected 28 h old cultured yeasts (10^6^ spores per ml of each fungal suspension, 100 µL/well) were moistened and spread on sterile potato dextrose agar (PDA) plates. Six wells 5 mm in diameter were punched in the center of the Petri plates by using a sterile cork borer. Different concentrations of JPEO from (125 to 1000 μg/mL; 100 µL/well) were added to each well. The plates were kept in a refrigerator for 30 min at 4 °C to allow JPEO to diffuse into the agar medium. The plates were finally incubated at 37 °C for 48 h. Fluconazole (25 μg/mL) was used as a positive control, whereas 1% DMSO with nutrient broth was used as a negative control. After overnight incubation, the ZOI diameters around JPEO were measured in mm.

#### 4.4.5. Determination of the MIC and MFC of JPEO

The MIC and MFC of JPEO were determined for all the fungal strains through the broth microdilution assay method in triplicate according to [54], with modifications. Briefly, Sabouraud dextrose broth (SDB) medium was prepared with increasing concentrations of JPEO (i.e., 1.95 to 1000 μg/mL) dissolved in 1% DMSO with SDB and added to each well. The selected yeasts (2.5 × 10^3^ fungal CFU/mL, 10 µL/well) were added to each well. Fluconazole (25 μg/mL) was used as a positive control, whereas 1% DMSO with free serum was used. After 24 h of incubation at 37 °C, the viability of the selected yeasts was assessed by adding 50 µL of TTC (2 mg/mL in PBS) working solution to each well, and the plates were incubated for 24 h at 37 °C. The well containing the lowest concentration of colorless solution was interpreted as the MIC, whereas the lowest concentration of JPEO at which no growth was recorded was the MFC.

### 4.5. Cell Culture and Cytotoxicity Assays

The cell lines MCF-7 (ATCC HTB-22) and HepG2 (ATCC HB-8065) were cultivated in DMEM supplemented with fetal calf serum and 1% penicillin–streptomycin at 37 °C in a humidified atmosphere with 5% CO_2_. The MTT assay was used to evaluate the cytotoxicity of JPEO against MCF-7 and HepG2 cells. This technique depends on mitochondrial dehydrogenases converting the MTT reagent to formazan crystals. In conclusion, 24 h before treatment, exponentially developing cells were collected with 0.25% trypsin-EDTA and plated in 96-well microplates with fresh complete medium at a density of 1103 1 × 103 cells/well (100 μL). For a whole day at 37 °C in a humidified atmosphere with 5% CO_2_, the cells were exposed to progressively higher concentrations of JPEO (50, 100, 200, and 400 μg/mL). Consequently, a 100 L mixture was produced. The positive control was cisplatin (30 µg/mL). Following the incubation periods, 10 µL of a working solution of MTT (5 mg/mL in phosphate-buffered saline, or PBS) was added to each well. The wells were then incubated for 4 h at 37 °C. Once the formazan particles had formed, they were dissolved in 100 μL/well of DMSO and agitated gently for 10 min at 37 °C. Finally, an automatic microplate reader (Biotek, Shoreline, WA, USA) known as ELX-808 was used to measure the absorbance of each well at 590 nm. The cell viability (%) was calculated as [(A − B)/A] × 100, where A and B represent the absorbances of treated cells and untreated control cells, respectively. Using Graph Pad Prism, the half-maximal inhibitory concentration (IC_50_) was calculated.

### 4.6. Antioxidant Activity

#### 4.6.1. DPPH Assay

Using a previously reported method, the ability of JPEO to react with DPPH radicals was evaluated via a DPPH radical scavenging assay [55]. The extracts were prepared at five different concentrations: 50, 100, 200, 400, and 800 μg/mL. For each concentration, approximately 0.5 mL of the extract was mixed with 0.375 mL of methanol and a DPPH solution (2 mL, 0.08 mM). The reaction mixture was then incubated in a dark environment for half an hour. Following incubation, the optical density (OD) of the combination was measured at 517 nm via a spectrophotometer (U2001 UV–vis Spectrophotometer, Hitachi, Japan). Ascorbic acid was used as a positive control at predetermined concentrations (400 µg/mL). The proportion of DPPH scavenging activity was determined via the following formula: DPPH radical scavenging activity [%] = [(Ac − A)/Ac] × 100, where Ac is the absorbance of the control, and A is the absorbance of the sample.

Using Graph Pad Prism software (version 5.0, La Jolla, CA, USA), the IC_50_ value was determined. The IC_50_ of DPPH scavenging activity was visually assessed via a linear equation that was generated from the concentration value and the percentage of antioxidant activity displayed on a graph, with the concentration value on the X-axis and the percentage activity on the Y-axis.

#### 4.6.2. ABTS Assay

The procedure outlined in a previous study [56] was followed to conduct the ABTS radical scavenging experiment. Ascorbic acid (200 μL, 400 μg/mL) was used as the positive control. To conduct the test, the K_2_S_2_O8 solution (140 mM) and the ABTS solution (192 mg/50 mL) were first mixed together. As a result, the reaction mixture was left in the dark at room temperature for around twelve hours. An OD of 0.70 ± 0.02 at 734 nm was also obtained by mixing the ABTS solution with methanol. Furthermore, 3 mL of diluted ABTS was thoroughly combined with 50 µL of each extract. Additionally, the mixture was incubated for six minutes in a dark environment. Next, a spectrophotometer (U2001 UV–vis Spectrophotometer, Hitachi, Japan) was used to detect the OD at 734 nm. The results are displayed as IC_50_ and ABTS% values.

### 4.7. Statistical Analysis

All experiments were performed in triplicate, and the results are expressed as the mean ± SD. Statistical analyses were conducted using GraphPad Prism software (version 5.0, GraphPad Software, San Diego, CA, USA). For antioxidant and cytotoxicity assays (Figure 2 and Figure 3), comparisons between treated groups and the corresponding control groups were performed using Student’s *t*-test. For antimicrobial and antifungal assays (Table 2 and Table 3), statistical differences among groups were analyzed using one-way analysis of variance (ANOVA) followed by Dunnett’s post hoc test, with comparisons made against the positive control. A value of *p* < 0.05 was considered statistically significant.

## 5. Conclusions

Research has focused on the JPEO, presenting a novel chemical profile rich in terpenoids with proven antibacterial and cytotoxic activities in vitro. The ethnopharmacological interest of this plant is supported by its distinct chemical profile and significant biological activity, placing it on the list of promising plants with potential for use in future pharmacological research. To translate existing leads into real therapeutic leads, a research program that can address existing lacunae, such as the mechanism of activity, safety, and viability, is essential.

## Figures and Tables

**Table 1:** Gas chromatography–mass spectrometry compounds in *Jacquemontia pentantha* essential oil.

Peak	RT	Area%	Name	MF	MW (g/mol)	Classification
1	5.587	2.08	o-Cymene	C_10_H_14_	134	Cumene
2	5.726	5.26	Eucalyptol	C_10_H_18_O	154	Oxane
3	6.053	0.29	(Z)-Sabinene hydrate	C_10_H_18_O	154	Monoterpenoid
4	6.172	0.19	Benzene, 1-methyl-4-(1-methylethenyl)-	C_10_H_12_	132	Phenylpropene
5	6.362	3.28	Thujone	C_10_H_16_O	152	Monoterpenoid
6	6.542	6.72	α-Thujone	C_10_H_16_O	152	Monoterpenoid
7	6.688	0.47	iso-3-Thujanol	C_10_H_18_O	154	Monoterpenoid
8	6.797	5.31	(+)-2-Bornanone	C_10_H_16_O	152	Monoterpenoid
9	6.907	1.42	Camphor	C_10_H_18_O	154	Monoterpenoid
10	6.97	0.56	L-4-terpineol	C_10_H_18_O	154	Monoterpenoid
11	7.013	0.25	o-Acetyltoluene	C_9_H_10_O	134	Carbonyl compound
12	7.062	0.44	L-α-Terpineol	C_10_H_18_O	154	Monoterpenoid
13	7.132	0.80	1,2,2,3-Tetramethylcyclopent-3-enol	C_9_H_16_O	140	Tertiary alcohol
14	7.425	0.29	Carvacrol methyl	C11H_16_O	164	Monoterpenoid
15	7.781	2.80	Carvacrol	C_10_H_14_O	150	Monoterpenoid
16	7.879	3.30	Thymol	C_10_H_14_O	150	Monoterpenoid
17	8.3	0.19	cis-Thujopsene	C_15_H_24_	204	Sesquiterpenoid
18	8.38	0.53	cis, cis-Nepetalactone	C_10_H_14_O_2_	166	Terpene lactone
19	8.454	0.32	α-Copaene	C_15_H_24_	204	Sesquiterpenoid
20	8.523	1.01	Isolongifolene, 4,5-dehydro-	C_15_H_22_	202	Polycyclic hydrocarbon
21	8.614	1.58	4-(2,4,4-Trimethyl-cyclohexa-1,5-dienyl)-but-3-en-2-one	C_13_H_18_O	190	Carbonyl compound
22	8.658	0.89	1H-Cyclopropa[a]naphthalene, 1a,2,3,3a,4,5,6,7b-octahydro-1,1,3a,7-tetramethyl-, [1aR-(1aα,3aα,7bα)]-	C_15_H_24_	204	Sesquiterpenoid
23	8.721	0.94	Longifolene	C_15_H_24_	204	Sesquiterpenoid
24	8.821	4.06	Caryophyllene	C_15_H_24_	204	Sesquiterpenoid
25	8.93	1.58	Aromandendrene	C_15_H_24_	204	Sesquiterpenoid
26	9.055	6.46	(+)-α-himachalene	C_15_H_24_	204	Sesquiterpenoid
27	9.156	0.90	γ-Himachalene	C_15_H_24_	204	Sesquiterpenoid
28	9.226	4.24	Longifolene-(V4)	C_15_H_24_	204	Monoterpenoid
29	9.289	0.60	Epicubebol	C_15_H_26_O	222	Sesquiterpenoid
30	9.379	6.47	β-Himachalene	C_15_H_24_	204	Sesquiterpenoid
31	9.427	3.54	α-Dehydro-ar-himachalene	C_15_H_20_	200	Benzenoid
32	9.525	2.13	γ-Dehydro-ar-himachalene	C_15_H_20_	200	Benzenoid
33	9.563	1.44	aR-Himachalene	C_15_H_22_	202	Benzenoid
34	9.629	0.53	2(1H)Naphthalenone, 3,5,6,7,8,8a-hexahydro-4,8a-dimethyl-6-(1-methylethenyl)-	C_15_H_22_O	218	Sesquiterpenoid
35	9.703	1.67	Murolan-3,9(11)-diene-10-peroxy	C_15_H_24_O_2_	236	Sesquiterpenoid
36	9.752	2.01	Aristol-1(10)-en-9-ol	C_15_H_24_O	220	Sesquiterpenoid
37	9.804	1.05	Dehydrofukinone	C_15_H_22_O	218	Sesquiterpenoid
38	9.89	2.59	Caryophyllene oxide	C_15_H_24_O	220	Sesquiterpenoid
39	9.964	3.45	Ledol	C_15_H_26_O	222	Sesquiterpenoid
40	10.069	2.69	(+)-β-Himachalene oxide	C_15_H_24_O	220	Epoxide
41	10.113	1.26	Calarene epoxide	C_15_H_24_O	220	Monoterpenoid
42	10.165	1.79	Isoaromadendrene epoxide	C_15_H_24_O	220	Sesquiterpenoid
43	10.28	1.47	α-acorenol	C_15_H_26_O	222	Tertiary alcohol
44	10.346	1.05	Bicyclo[4.4.0]dec-2-ene-4-ol, 2-methyl-9-(prop-1-en-3-ol-2-yl)-	C_15_H_24_O_2_	236	Sesquiterpenoid
45	10.378	1.19	α-Bisabolol	C_15_H_26_O	222	Sesquiterpenoid
46	10.504	0.88	(Z)-α-Atlantone	C_15_H_22_O	218	Sesquiterpenoid
47	10.562	1.73	β-Guaiene	C_15_H_24_	204	Sesquiterpenoid
48	10.712	0.72	Ylangenal	C_15_H_22_O	218	Sesquiterpenoid
49	10.769	1.72	(E)-Atlantone	C_15_H_22_O	218	Sesquiterpenoid
50	10.896	1.33	6-Isopropenyl-4,8a-dimethyl-1,2,3,5,6,7,8,8a-octahydronaphthalene-2,3-diol	C_15_H_24_O_2_	236	Sesquiterpenoid
51	11.158	0.55	2(1H)Naphthalenone, 3,5,6,7,8,8a-hexahydro-4,8a-dimethyl-6-(1-methylethenyl)-	C_15_H_22_O	218	Sesquiterpenoid
52	11.276	0.34	6-(1-Hydroxymethylvinyl)-4,8a-dimethyl-3,5,6,7,8,8a-hexahydro-1H-naphthalen-2-one	C_15_H_22_O_2_	234	Sesquiterpenoid
53	11.732	0.21	Isopimara-9(11),15-diene	C_20_H_32_	272	Diterpenoid
54	11.826	0.09	Cupressene	C_20_H_32_	272	Diterpenoid
55	12.189	0.10	Androstan-17-one, 3-ethyl-3-hydroxy-, (5α)-	C_21_H_34_O_2_	318	Androstane steroid
56	12.437	1.13	13-Epimanool	C_20_H_34_O	290	Diterpenoid
57	12.605	0.11	Eudesm-11-en-1-ol	C_15_H_26_O	222	Sesquiterpenoid
Totals			Sesquiterpenoids			45.1%
			Monoterpenoids			30.4%
			Benzenoids			7.1%
			Oxanes			5.3%
			Others			12.1%

Molecular formulae and molecular weights were obtained from reference databases [14] and were not determined experimentally in this study.

**Table 2:** Zone of inhibition, minimum inhibitory concentration, and minimum bactericidal concentration of *Jacquemontia pentantha* essential oil.

Bacterium/Dilution	Positive Control	400 μg/mL	200 μg/mL	100 μg/mL	50 μg/mL	MIC (μg/mL)	MBC (μg/mL)
*Staphylococcus aureus*	27 ± 1.68	19 ± 2.61 *	17 ± 0.95 *	14 ± 0.67 *	11 ± 0.29 *	25 ± 0.00	50 ± 0.00
*Staphylococcus epidermidis*	24 ± 1.55	20 ± 1.98 *	16 ±1.36 *	12 ± 1.92 *	10 ± 1.73 *	6.25 ± 0.00	12.50 ± 0.00
*Enterococcus faecalis*	26 ± 2.46	19 ± 1.39 *	17 ± 1.97 *	14 ± 1.55 *	11 ± 1.43 *	6.25 ± 0.00	12.50 ± 0.00
*Escherichia coli*	25 ± 2.17	23 ± 2.55 *	20 ± 1.26 *	18 ± 1.48 *	14 ± 2.34 *	9.357 ± 4.14	12.5 ± 0.00
*Klebsiella pneumoniae*	25 ± 1.35	22 ± 1.46 *	19 ± 0.94 *	17 ± 1.37 *	15 ± 1.74 *	12.50 ± 0.00	25 ± 0.00
*P. aeruginosa*	24 ± 0.24	23 ± 0.75 *	21 ± 1.37 *	19 ± 1.26 *	14 ± 2.44 *	4.68 ± 2.21	6.25 ± 0.00

Note zone of inhibition (ZOI), minimum inhibitory concentration (MIC), and minimum bactericidal concentration (MBC). Statistical analysis was performed via one-way ANOVA and post hoc test. The reported values are shown in triplicate as the means ± SDs. The results revealed a statistically significant decrease from the positive control (25 µg/mL chloramphenicol), as indicated by * = *p* < 0.05.

**Table 3:** Zone of inhibition, minimum inhibitory concentration, and minimum fungicidal concentration of *Jacquemontia pentantha* essential oil.

Fungal/Dilution	Positive Control	1000 μg/mL	500 μg/mL	250 μg/mL	125 μg/mL	MIC (μg/mL)	MFC (μg/mL)
*Candida albicans*	20 ± 0.00	25 ± 0.00	19 ± 0.00 *	16 ± 0.00 *	12 ± 0.00 *	7.81 ± 0.88	3.90 ± 0.68
*Candida glabrata*	22 ± 0.00	19 ± 0.00 *	12 ± 0.00 *	9 ± 0.00 *	6 ± 0.00 *	250 ± 9.98	125 ± 11.61
*Candida kefyr*	33 ± 0.00	37 ± 0.00	32 ± 0.00 *	19 ± 0.00 *	13 ± 0.00 *	15.62 ± 4.21	7.81 ± 3.51
*Candida parapsilosis*	23 ± 0.00	14 ± 0.00 *	12 ± 0.00 *	8 ± 0.00 *	6 ± 0.00 *	62.5 ± 5.22	31.25 ± 6.54
*Candida tropicalis*	25 ± 0.00	22 ± 0.00 *	13 ± 0.00 *	10 ± 0.00 *	7 ± 0.00 *	62.5 ± 4.23	31.25 ± 5.46

Note zone of inhibition (ZOI), minimum inhibitory concentration (MIC), and minimum fungicidal concentration (MFC). Statistical analysis was performed via one-way ANOVA and post hoc test. The reported values are shown in triplicate as the means ± SDs. The results revealed a statistically significant decrease from the positive control (25 µg/mL, fluconazole), as indicated by * = *p* < 0.05.

## Data Availability

Data is contained within the article.

## References

[1] Bunse M., Daniels R., Gründemann C., Heilmann J., Kammerer D.R., Keusgen M., Lindequist U., Melzig M.F., Morlock G.E., Schulz H. (2022). Essential oils as multicomponent mixtures and their potential for human health and well-being. Front. Pharmacol..

[2] El Boukhari R., Fatimi A. (2025). The Innovative potential of key mentha species: An assessment based on patent analysis. Biol. Life Sci. Forum.

[3] El-Saadony M.T., Saad A.M., Mohammed D.M., Korma S.A., Alshahrani M.Y., Ahmed A.E., Ibrahim E.H., Salem H.M., Alkafaas S.S., Saif A.M. (2025). Medicinal plants: Bioactive compounds, biological activities, combating multidrug-resistant microorganisms, and human health benefits-a comprehensive review. Front. Immunol..

[4] Modi N., Dhokiya M., Kadia R., Dabhi M., Goswami D. (2025). Bioactive compounds and biological activities of *Jacquemontia Pentanthos* (Jacq.) G. Don.: A Phytochemical and pharmacological study. Int. J. Sci. Res. Technol..

[5] Eskander D.M., El-Khrisy E.-D.A., Grace M.H., Nabil M., Nassar M.I., Mounier M.M. (2019). Investigation of secondary metabolites and cytotoxicity of *Jacquemontia pentantha* (Jacq.). Pharmacogn. J..

[6] Zahedi S.M., Karimi M., Venditti A. (2021). Plants adapted to arid areas: Specialized metabolites. Nat. Prod. Res..

[7] Magwede K., Van Wyk B.-E., Van Wyk A.E. (2019). An inventory of vhavenḓa useful plants. S. Afr. J. Bot..

[8] Zhao H., Ren S., Yang H., Tang S., Guo C., Liu M., Tao Q., Ming T., Xu H. (2022). Peppermint essential oil: Its phytochemistry, biological activity, pharmacological effect and application. Biomed. Pharmacother..

[9] Hudz N., Kobylinska L., Pokajewicz K., Horčinová Sedláčková V., Fedin R., Voloshyn M., Myskiv I., Brindza J., Wieczorek P.P., Lipok J. (2023). Mentha piperita: Essential oil and extracts, their biological activities, and perspectives on the development of new medicinal and cosmetic products. Molecules.

[10] Kazemi A., Iraji A., Esmaealzadeh N., Salehi M., Hashempur M.H. (2025). Peppermint and menthol: A review on their biochemistry, pharmacological activities, clinical applications, and safety considerations. Crit. Rev. Food Sci. Nutr..

[11] Ailli A., Handaq N., Touijer H., Gourich A.A., Drioiche A., Zibouh K., Eddamsyry B., El Makhoukhi F., Mouradi A., Bin Jardan Y.A. (2023). Phytochemistry and biological activities of essential oils from six aromatic medicinal plants with cosmetic properties. Antibiotics.

[12] Bibow A., Oleszek W. (2024). Essential oils as potential natural antioxidants, antimicrobial, and antifungal agents in active food packaging. Antibiotics.

[13] Parvathy C., Lohidas J. (2025). First report on the phytochemical, nutritional and antioxidant potential of *Jacquemontia pentanthos*, a key plant species from Kerala, India. Plant Sci. Today.

[14] Kim S., Chen J., Cheng T., Gindulyte A., He J., He S., Li Q., Shoemaker B.A., Thiessen P.A., Yu B. (2023). PubChem 2023 update. Nucleic Acids Res..

[15] Lee S.T., Gardner D.R., Cook D. (2017). Identification of indole diterpenes in *Ipomoea asarifolia* and *Ipomoea muelleri*, plants tremorgenic to livestock. J. Agric. Food Chem..

[16] Salamatullah A.M. (2022). Convolvulus arvensis: Antioxidant, antibacterial, and antifungal properties of chemically profiled essential oils: An approach against nosocomial infections. Life.

[17] Giuliani C., Bottoni M., Ascrizzi R., Milani F., Spada A., Flamini G., Fico G. (2021). Morphology and phytochemistry of *Teucrium chamaedrys* L. (*Lamiaceae*) cultivated at the Ghirardi Botanic Garden (Lombardy, Northern Italy). Flora.

[18] Giuliani C., Bottoni M., Ascrizzi R., Milani F., Spada A., Papini A., Flamini G., Fico G. (2023). Insight into micromorphology and phytochemistry of *Lavandula angustifolia* Mill. from Italy. S. Afr. J. Bot..

[19] Zámboriné Németh É., Thi Nguyen H. (2020). Thujone, a widely debated volatile compound: What do we know about it?. Phytochem. Rev..

[20] Furtado A.A., Torres-Rêgo M., Lima M.C., Bitencourt M.A., Estrela A.B., da Silva N.S., da Silva Siqueira E.M., Tomaz J.C., Lopes N.P., Silva-Júnior A.A. (2016). Aqueous extract from *Ipomoea asarifolia* (*Convolvulaceae*) leaves and its phenolic compounds have anti-inflammatory activity in murine models of edema, peritonitis and air-pouch inflammation. J. Ethnopharmacol..

[21] Dinku W., Park S.B., Jeong J.B., Jung C., Dekebo A. (2022). Chemical composition and anti-inflammatory activity of essential oils from resin of *Commiphora* species. Bull. Chem. Soc. Ethiop..

[22] Gushiken L.F.S., Beserra F.P., Hussni M.F., Gonzaga M.T., Ribeiro V.P., De Souza P.F., Campos J.C.L., Massaro T.N.C., Hussni C.A., Takahira R.K. (2022). Beta-caryophyllene as an antioxidant, anti-inflammatory and re-epithelialization activities in a rat skin wound excision model. Oxidative Med. Cell. Longev..

[23] Dickson K., Scott C., White H., Zhou J., Kelly M., Lehmann C. (2023). Antibacterial and analgesic properties of beta-caryophyllene in a murine urinary tract infection model. Molecules.

[24] Mendoza-Almanza G., Esparza-Ibarra E.L., Ayala-Luján J.L., Mercado-Reyes M., Godina-González S., Hernández-Barrales M., Olmos-Soto J. (2020). The cytocidal spectrum of *Bacillus thuringiensis* toxins: From insects to human cancer cells. Toxins.

[25] Hernandez-Rojas A.C., Fragoso-Serrano M., Pereda-Miranda R. (2025). The jalap roots: A herbal legacy from the neotropics to the world. J. Ethnopharmacol..

[26] Alqethami A., Aldhebiani A.Y. (2021). Medicinal plants used in Jeddah, Saudi Arabia: Phytochemical screening. Saudi J. Biol. Sci..

[27] Saraux N., Imeri D., Quirós-Guerrero L., Karimou S., Christen P., Cuendet M. (2021). Phytochemical investigation of the roots of *Ipomoea asarifolia* and Antiproliferative activity of the isolated compounds against multiple myeloma cells. J. Nat. Prod..

[28] Wołosiak R., Drużyńska B., Derewiaka D., Piecyk M., Majewska E., Ciecierska M., Worobiej E., Pakosz P. (2021). Verification of the conditions for determination of antioxidant activity by ABTS and DPPH assays—A practical approach. Molecules.

[29] Baliyan S., Mukherjee R., Priyadarshini A., Vibhuti A., Gupta A., Pandey R.P., Chang C.-M. (2022). Determination of antioxidants by DPPH radical scavenging activity and quantitative phytochemical analysis of *Ficus religiosa*. Molecules.

[30] Alagesan V., Ramalingam S., Kim M., Venugopal S. (2019). Antioxidant activity guided isolation of a coumarin compound from *Ipomoea pes-caprea* (*Convolvulaceae*) leaves acetone extract and its biological and molecular docking studies. Eur. J. Integr. Med..

[31] Gulcin İ., Alwasel S.H. (2023). DPPH radical scavenging assay. Processes.

[32] Pang Z., Raudonis R., Glick B.R., Lin T.-J., Cheng Z. (2019). Antibiotic resistance in *Pseudomonas aeruginosa*: Mechanisms and alternative therapeutic strategies. Biotechnol. Adv..

[33] Hu M., Chua S.L. (2025). Antibiotic-Resistant *Pseudomonas aeruginosa:* Current challenges and emerging alternative therapies. Microorganisms.

[34] Hulankova R. (2024). Methods for Determination of antimicrobial activity of essential oils in vitro—A review. Plants.

[35] Swamy M.K., Akhtar M.S., Sinniah U.R. (2016). Antimicrobial properties of plant essential oils against human pathogens and their mode of action: An updated review. Evid.-Based Complement. Altern. Med..

[36] Silva W., Bona N., Pedra N., Cunha K.D., Fiorentini A., Stefanello F., Zavareze E., Dias A. (2022). Risk assessment of in vitro cytotoxicity, antioxidant and antimicrobial activities of *Mentha piperita* L. essential oil. J. Toxicol. Environ. Health Part A.

[37] Noshad Q., Ajaib M., Kiran A. (2023). Antioxidant and Antimicrobial activity of *Cuscuta reflexa* ROXB. and few members of family *Convolvulaceae*. Curr. Trends OMICS.

[38] Iskandar K., Ahmed N., Paudyal N., Ruiz Alvarez M.-J., Balasubramani S.P., Saadeh D., Ullah Baig S., Sami H., Hammoudi Halat D., Pavlović N. (2025). Essential Oils as antimicrobial agents against WHO Priority bacterial pathogens: A strategic review of in vitro clinical efficacy, innovations and research gaps. Antibiotics.

[39] Konuk H.B., Ergüden B. (2020). Phenolic–OH group is crucial for the antifungal activity of terpenoids via disruption of cell membrane integrity. Folia Microbiol..

[40] Franconi I., Lupetti A. (2023). In vitro susceptibility tests in the context of antifungal resistance: Beyond minimum inhibitory concentration in *Candida* spp.. J. Fungi.

[41] Hrichi S., Chaâbane-Banaoues R., Alibrando F., Altemimi A.B., Babba O., Majdoub Y.O.E., Nasri H., Mondello L., Babba H., Mighri Z. (2022). Chemical composition, antifungal and anti-biofilm activities of volatile fractions of *Convolvulus althaeoides* L. roots from Tunisia. Molecules.

[42] Armadany F.I., Sopyan I., Mustarichie R. (2024). Antifungal activity and hair growth stimulation of purple sweet potato leaf fraction (*Ipomoea batatas* (L.) Lamk) and its molecular mechanism through androgen receptor inhibition. Pharmacia.

[43] Ezzariga N., Moukal A., Asdadi A., Lemkhente Z., Moustaoui F., Kaaya A., Aghrouch M. (2025). Evaluation of the Antimicrobial activity of 20 essential oils and their combinations on bacterial and fungal strains. Cureus.

[44] Al-Khaial M.Q., Fahad M.M., Al-Khateeb F.N. (2025). Chemical composition, antioxidant capacity, and anticancer activity of the essential oil from aerial parts of *Convolvulus lanatus* Vahl. J. Pharm. Pharmacogn. Res..

[45] Tokgun O., Akca H., Mammadov R., Aykurt C., Deniz G. (2012). *Convolvulus galaticus*, *Crocus antalyensis*, and *Lilium candidum* extracts show their antitumor activity through induction of p53-mediated apoptosis on human breast cancer cell line MCF-7 cells. J. Med. Food.

[46] Hendel N., Sarri D., Sarri M., Napoli E., Palumbo Piccionello A., Ruberto G. (2024). Phytochemical Analysis and antioxidant and antifungal activities of powders, methanol extracts, and essential oils from *Rosmarinus officinalis* L. and *Thymus ciliatus Desf. Benth*. Int. J. Mol. Sci..

[47] Abbas A., Naqvi S.A.R., Rasool M.H., Noureen A., Mubarik M.S., Tareen R.B. (2021). Phytochemical analysis, antioxidant and antimicrobial screening of *Seriphidium oliverianum* plant extracts. Dose-Response.

[48] Wolfe K.L., Liu R.H. (2003). Apple peels as a value-added food ingredient. J. Agric. Food Chem..

[49] Ordonez A., Gomez J., Vattuone M. (2006). Antioxidant activities of *Sechium edule* (Jacq.) Swartz extracts. Food Chem..

[50] Salem N., Kefi S., Tabben O., Ayed A., Jallouli S., Feres N., Hammami M., Khammassi S., Hrigua I., Nefisi S. (2018). Variation in chemical composition of *Eucalyptus globulus* essential oil under phenological stages and evidence synergism with antimicrobial standards. Ind. Crops Prod..

[51] Basri D.F., Sandra V. (2016). Synergistic interaction of methanol extract from *Canarium odontophyllum* Miq. Leaf in combination with oxacillin against methicillin-resistant *Staphylococcus aureus* (MRSA) ATCC 33591. Int. J. Microbiol..

[52] Aljeldah M.M., Yassin M.T., Mostafa A.A.-F., Aboul-Soud M.A. (2022). Synergistic Antibacterial potential of greenly synthesized silver nanoparticles with fosfomycin against some nosocomial bacterial pathogens. Infect. Drug Resist..

[53] Liu Y., Tortora G., Ryan M.E., Lee H.-M., Golub L.M. (2002). Potato dextrose agar antifungal susceptibility testing for yeasts and molds: Evaluation of phosphate effect on antifungal activity of CMT-3. Antimicrob. Agents Chemother..

[54] Rangel M.d.L., Aquino S.G.d., Lima J.M.d., Castellano L.R., Castro R.D.d. (2018). In vitro effect of *Cinnamomum zeylanicum* Blume essential oil on Candida spp. involved in oral infections. *Evid.-Based Complement*. Altern. Med..

[55] Tian M., Wu X., Lu T., Zhao X., Wei F., Deng G., Zhou Y. (2020). Phytochemical analysis, antioxidant, antibacterial, cytotoxic, and enzyme inhibitory activities of *Hedychium flavum* rhizome. Front. Pharmacol..

[56] Yu X., Zhao M., Liu F., Zeng S., Hu J. (2013). Antioxidants in volatile Maillard reaction products: Identification and interaction. LWT-Food Sci. Technol..

